# Comment on Park et al. Lessons Learned from Translating Genome Sequencing to Clinical Routine: Understanding the Accuracy of a Diagnostic Pipeline. *Genes* 2024, *15*, 136

**DOI:** 10.3390/genes15101322

**Published:** 2024-10-14

**Authors:** Florian Battke, Martin Schulze, Björn Schulte, Saskia Biskup

**Affiliations:** 1 CeGaT GmbH, Paul-Ehrlich-Str. 23, 72076 Tuebingen, Germany; 2 Center for Human Genetics, Paul-Ehrlich-Str. 23, 72076 Tuebingen, Germany

In their article, “Lessons Learned from Translating Genome Sequencing to Clinical Routine: Understanding the Accuracy of a Diagnostic Pipeline”, Haack et al. [1] present data from the routine diagnostic use of genome and exome sequencing performed in 2022 in their institution and evaluate the benefit of using genome sequencing in such a setting. The authors show that their bioinformatics pipeline yields diagnostically relevant results from both data types, and they present technical information on different metrics and variant calling performance. They also compare the diagnostic yields of genome sequencing (GS) and exome sequencing (ES).

In this comment, we focus on the question of whether the present publication can help us understand the benefits of GS over ES. We do not question the author’s successful implementation of genome sequencing in routine clinical diagnostics.

The paper is conceptually divided into two parts. The first examines two cohorts of analyses, one performed using ES and one using GS. While this shows that both methods can be used in clinical practice, the cohorts are far too disparate to draw any comparative conclusions. The exomes were mostly of children (median age 7) and a significant portion consisted of trio analyses (22%), while the genomes were of middle-aged adults (median age 49) with hardly any trios performed (2%). The ES cohort was analyzed in 2022 using an off-the-shelf exome kit (Agilent Human All Exon version 7). Since then, newer enrichment kits and protocols brought about improvements in target coverage. Many institutions, including the authors’, have implemented custom-design exome enrichment to address some of the shortcomings of standard exome kits and optimize ES for clinical diagnostics. The figures presented on coverage dropouts in ES vs. GS should be interpreted with that in mind.

The second part of the paper uses the GS cohort to investigate whether this method improves diagnostic yield over ES. This approach has been used before and is a good method to show the benefits of the method. However, to be useful and in the furtherance of the scientific knowledge on this issue, comparisons must be fair, and conclusions drawn must be supported by the data. The authors present that of the 416 solved cases in GS, 37 were solved by variations that could not have been detected using ES. Unfortunately, the precise nature of those 37 variations was not part of the publication or supplement and could not be obtained from the authors upon request due to data privacy considerations.

We must note however, that of these 37 variations, 13 are variants in non-coding intronic regions. Exome kits optimized for clinical diagnostics routinely cover such variants if they are known or suspected to be disease-causing. A further 10 variants were small CNV covering up to three exons. CNV calling in exome data is more involved than in whole-genome data, but the higher coverage in exome sequencing results in good sensitivity for single exon deletions too. These two variant categories would warrant manual inspection in exome data to confirm the authors’ statements. Unfortunately, the article does not present the results of such inspections.

The other variant categories (12 repeat expansions and 2 structural variants) are less contentious, even though repeat expansion calling from exome data is possible and the main benefit of GS in this regard is the coverage of repeat expansion regions not covered in exome enrichment. Depending on which repeats were detected in this cohort, these variants may or may not have been detected by ES. While polymerase slippage may affect the precise number of repeat units detected in ES data, studies have already shown that the presence of pathogenic repeat expansions can be detected from ES data. There is no conclusive evidence showing that enrichment sequencing data is generally unsuited to repeat expansion calling.

Accepting that all 37 variants would truly be invisible to state-of-the-art diagnostic exome sequencing, one major problem remains; the authors compute the yield increase obtained by using GS instead of ES, stating that their ”data suggest an increase in diagnostic yield of almost 9%“. Given a cohort of 1977 patients analyzed Tusing GS, 37 additional solved cases out of a total of 416 solved cases represent an increase in yield from 19.2% to 21.0%. Anyone interested in the diagnostic yield increase would conclude that the yield increased by 1.8%, far off the claimed 9%. Technically, this is the difference between a relative increase of 9 percent and an absolute increase of 1.8 percentage points. While it would be correct to say that 9% of the solutions obtained by GS might not have been obtained using ES in this cohort, the authors’ presentation of this figure as the increase in yield is very misleading. And given that the detectability of CNVs and known disease-causing intronic mutations may not have been evaluated fairly against the current state of ES, the yield increase may well be much smaller.

We do not doubt that genome sequencing can increase diagnostic yield over exome sequencing. However, to make informed decisions, we need studies that provide meaningful data. The present manuscript muddies the waters by presenting data from two very disparate cohorts side by side, by the incomplete analysis of the variants supposedly only detectable by GS, and by the misleading statement on the possible yield increase. It would have been better to present a focused report of the authors’ experiences in their successful implementation of GS into clinical practice, and indeed share the lessons learned in that process, leaving aside questions of diagnostic yield which their datasets cannot answer.
